# Optical Modulation of the Diffraction Efficiency in an Indoline Azobenzene/Amorphous Polycarbonate Film

**DOI:** 10.1186/s11671-016-1542-2

**Published:** 2016-07-15

**Authors:** G. V. M. Williams, My T. T. Do, A. Middleton, S. G. Raymond, M. D. H. Bhuiyan, A. J. Kay

**Affiliations:** MacDairmid Institute, School of Chemical and Physical Sciences, Victoria University of Wellington, PO Box 600, Wellington, 6012 New Zealand; Callaghan Innovation, PO Box 31310, Lower Hutt, 5040 New Zealand

**Keywords:** Diffraction grating, Photochromic, Indoline azobenzene, Thin film

## Abstract

We have made a diffraction grating in an indoline azobenzene/amorphous polycarbonate film by two-beam interference at 532 nm that periodically photodegrades the indoline azobenzene dye. Subsequent illumination of the film with 532-nm light into the *trans*-isomer band leads to *trans*-*cis* isomerization in the indoline azobenzene dye and results in a decrease in the *trans*-isomer band absorption coefficient. This causes the diffraction efficiency to decrease when probed at 655 nm. The diffraction efficiency returns to its original value when the 532-nm light is blocked by thermal relaxation from the indoline azobenzene *cis*-isomer to the *trans*-isomer. Thus, we have been able to optically modulate the diffraction efficiency in a thin film diffraction grating.

## Background

Optical filters based on diffraction gratings have a large range of applications that include optical storage [1, 2], optical communication [3–5], strain and chemical sensing [6–9], and spectroscopy [10–12]. They have been made and researched using different methods and materials. For example, doped photorefractive LiNbO_3_ single crystals have been used to make optical filters where they are made permanent by thermal fixing [3, 13]. Optical filters have also been produced using liquid crystals [14–16], porous silicon [9, 12], anodic aluminina [17], polymers [4, 18], photochromic films [19, 20], nonlinear optical chromophore thin films [21], and RbCdF_3_:Mn^2+^ single crystals [22]. Optically switchable diffraction gratings would be particularly useful for all-optical switching and multiplexing [5] and could be used, for example, in add/drop [4] modules in optical communication systems that are needed for dense wavelength division multiplexing.

We have recently shown that an optically switchable diffraction grating can be made using a photochromic dye/amorphous polycarbonate (APC) composite film. In that case, the dye used was 5-chloro-1,3-dihydro-1,3,3-trimethylspiro[2H-indole-2,3′-(3H)naphth[2,1-b](1,4)oxazine] where UV light is required to break a band that results in a visible absorption band [23]. It was shown that a diffraction grating could be made by two-beam interference, the grating could subsequently be turned on by UV irradiation, and it could disappear after the UV light is turned off. This method does require the use of UV light to turn the diffraction grating on, and it would be advantageous if another dye is used that only requires more easily accessible visible or infrared light.

In this paper, we report the results from optical measurements on a film containing an indoline azobenzene dye in an APC matrix where a two-beam interference method was used to create a diffraction grating. We show that a diffraction grating can be made and the diffraction efficiency can be reduced by exposure to visible light and the recovery of the grating occurs by thermal relaxation.

## Methods

A photochromic dye/polymer thin film was made using APC and an indoline azobenzene (IAB) dye. The APC used was APEC 9389, and it was obtained from Bayer Materials Science. IAB was synthesized via coupling of the diazonium salt of 4-aminobenzonitrile to 1,3,3-trimethyl-2-methylene indoline using standard conditions. The product was obtained as a red solid, and it was purified by recrystallization in ethanol. An IAB/APC thin film was prepared that contained 5 % of IAB by weight. This was done by dissolving 50 mg of the dye and 950 mg of APC in 10 mL of 1,1,2-trichloroethane (TCE) and stirring the solution for 30 min at ~40 °C. The solution was then passed through 0.45- and then 0.22-μm syringe filters. The solution was spin-coated onto 25 × 25 mm^2^ glass substrates to create the thin film that was dried in a vacuum in the dark at 80 °C for several days. To minimize the risk of any unintended photo-switching, the dried films were kept in the dark until required.

An Ocean Optics spectrometer or a PerkinElmer Lambda 1050 spectrophotometer was used to make the optical measurements. The film was 2.6-μm thick as estimated from the resultant thin film interference. The optically induced changes in the IAB absorbance were measured by placing a cuvette containing a IAB/TCE solution in the Ocean Optics spectrometer. An initial absorbance measurement was made, and then the solution was exposed to an expanded 532-nm laser beam from a 5-W diode-pumped solid-state frequency-doubled Nd:YVO_4_ laser. This was immediately followed by an absorbance measurement. The photo-induced changes in the absorption coefficient from the IAB/APC film at 488 nm were measured using the 488-nm line from an Ar ion laser. The beam was split into two beams, and the probe beam went through a 1 % neutral density filter.

A diffraction grating was made in the IAB/APC film by using a cube beam splitter to split the 532-nm laser beam into two beams that overlapped on the film surface. The first-order diffracted beam from one of the beams was used to monitor the resultant diffraction efficiency. The angle between the beams was 3.2°. The diffraction efficiency was also probed at 655 nm where there is no absorption from the IAB dye using a 10-mW diode laser where the incident intensity was 5 mW. Silicon photodiodes were used to measure the transmitted and diffracted beam intensities.

## Results and Discussion

**Fig. 1:**
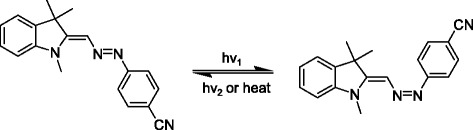
The indoline azobenzene *trans*-isomer (*left*) and *cis*-isomer (*right*). *Trans*-*cis* isomerization can occur by optical excitation into the *trans*-isomer absorption band. The reverse process can occur thermally or by optical excitation into the *cis*-isomer absorption band

Photo-switching of IAB occurs by a *trans*-*cis* isomerization process as can be seen in Fig. 1 where the *trans*- and *cis*-isomer structures are shown. Exposure of the *trans*-isomer (left structure) to visible light results in *trans*- to *cis*-isomerization (right structure). The *cis*-isomer can thermally relax back to the *trans*-isomer or this process can occur via optical excitation. This *trans*-*cis* isomerization process is well-known in a number of photochromic dyes including the azobenzene dyes [24, 25].

**Fig. 2:**
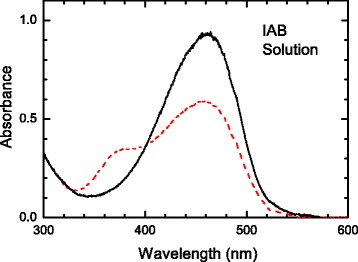
Plot of the absorbance from an indoline azobenzene solution in 1,1,2-trichloroethane (*solid curve*). Also shown is the absorbance during illumination with 532-nm laser light (*dashed curve*)

The absorbance of a TCE solution containing IAB is shown in Fig. 2. There is an absorption maximum at ~470 nm (solid curve) from the *trans*-isomer. Exposure to laser light at 532 nm led to a reduction in the ~470 nm *trans*-isomer absorption band and the appearance of a second peak at ~370 nm (dashed curve) from the *cis*-isomer.

**Fig. 3:**
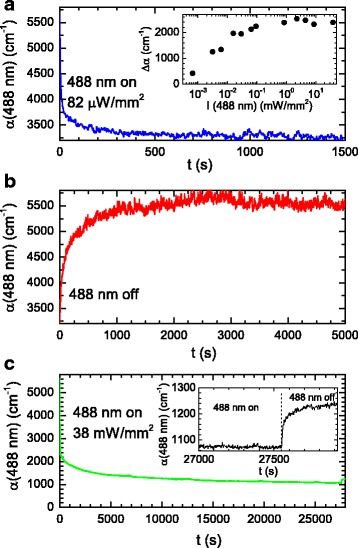
**a** Plot the absorption coefficient against time during bleaching of a 5 % IAB/APC film at 488 nm with an incident intensity of 82 μW/mm^2^. *Inset*: plot of the maximum change in the absorption coefficient at 488 nm against the 488-nm intensity. **b** Plot of the absorption coefficient against time for a 5 % IAB/APC film at 488 nm after the bleaching beam at 488 nm was switched off. **c** Plot of the absorption coefficient against time during bleaching and photodegradation of a 5 % IAB/APC film at 488 nm for an incident intensity of 38 mW/mm^2^. The bleaching beam was blocked after 27,550 s, and there is a small increase in the absorption coefficient that is more clearer in the inset

An IAB/APC film containing 5 % IAB by weight was exposed to 488-nm light, and the resulting change in the absorption coefficient, *α*, at 488 nm can be seen in Fig. 3a for a 488-nm switching intensity of 82 μW/mm^2^ and when probed with a weak intensity 488-nm probe beam. This wavelength was chosen because it is close to the *trans*-isomer peak absorption coefficient; hence, photo-induced changes in *α* from *trans*-*cis* isomerization are expected to be faster than that which could occur if a longer wavelength was used. The time for the absorption coefficient to decay by 1/*e* was ~11 s. We show in the inset of Fig. 3a that the change in *α* increases with increasing intensity and it saturates at high 488-nm intensities. The saturation is likely to be due to equilibrium between optically induced *trans*- to *cis*-isomerization by exciting into the *trans*-isomer band peaked at ~470 nm and thermal relaxation from the *cis*- to the *trans*-isomer.

Thermal relaxation can be seen in Fig. 3b when the 488-nm switching laser light was blocked. The 1/*e* thermal relaxation time was ~116 s. Photodegradation occurs for high intensities, as can be seen in Fig. 3c for a 488-nm switching beam intensity of 38 mW/mm^2^. The film containing IAB initially switches, and then there is a gradual decrease in the absorption coefficient. The switching beam was blocked after 27,550 s, and it can be seen that the absorption coefficient increased by only 170 cm^−1^ after this time and it does not increase back to the initial value. This shows that there is photodegradation of IAB.

The photodegradation process during high-intensity 488-nm illumination is likely to be oxygen-mediated and similar to that seen in other organic compounds [26–28]. Optical excitation leads to an excited singlet state, and relaxation occurs via a transition to the singlet ground state as well as intersystem crossing to the triplet ground state that leads to the generation of singlet oxygen [26–28]. The transition from the ground state triplet to the lower energy ground singlet state is spin-forbidden, but it can occur in the presence of triplet oxygen and results in the generation of singlet oxygen. It is the singlet oxygen that chemically interacts with dye and leads to the loss of the visible absorption band. This is a well-known problem in organic dyes, and it can be significantly reduced by encapsulation as well as by using singlet oxygen quenchers [28–31].

We have shown above that photo-induced changes in *α* can be made in a film containing IAB. For low intensities and times, these changes are nearly reversible and there is minimal photodegradation of IAB. However, there is significant photodegradation for high intensities, which can be exploited to create diffraction gratings as shown below.

Diffraction gratings were made by two-beam interference that leads to a sinusoidal optical intensity in the film with the modulation direction being parallel to the film surface. This results in a periodic modulation of the absorption coefficient via *trans*- to *cis*-isomerization as well as gradual photodegradation of IAB. A modulation of the absorption coefficient also means that there will be a periodic modulation of the refractive index. In the thin film limit and for small photo-induced changes in the absorption coefficient, the complex refractive index modulation can be written as *Δñ* = [*Δn*/2 + *i*(*λ*_probe_/(2*πn*))(*Δα*/2)]sin(*Λx*), where Δ*n* is the maximum change in the refractive index, Δ*α* is the maximum change in the absorption coefficient, *λ*_probe_ is the probe wavelength, *n* is the average refractive index, Λ is the diffraction grating period, and *x* is the modulation direction that is parallel to the film surface. For small angle and weak diffraction of a probe beam at normal incidence, the first-order diffraction efficiency in the thin limit is [32],

1 $$ {\eta}_1= \exp \left(-2{\alpha}_0z\right)\left[{\left(\frac{\pi \varDelta nz}{2{\lambda}_{\mathrm{probe}}}\right)}^2+{\left(\frac{\varDelta \alpha z}{4}\right)}^2\right] $$

where *η*_1_ is the first-order beam intensity divided by the zeroth-order beam intensity, *α*_0_ is the absorption coefficient, and *z* is the film thickness.

**Fig. 4:**
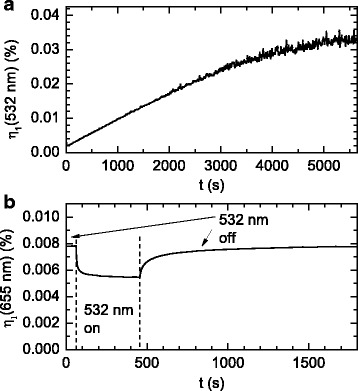
**a** Plot of first order diffraction efficiency at 532 nm from the 5 % IAB/APC film during two-beam grating creation at 532 nm. The intensity from each beam was 3.2 mW/mm^2^. **b** Plot of the first order diffraction efficiency at 655 nm after the grating was created and when there is a single 532-nm beam intensity of 111 μW/mm^2^ between 61 and 455 s (the *region* between the *vertical dashed lines*)

We show in Fig. 4a the first-order diffraction efficiency at 532 nm for the IAB/APC film during two-beam grating creation at 532 nm and with an intensity of 3.2 mW/mm^2^ from each beam. This wavelength was used to create the grating because the 1/*e* absorption depth into the film (19 μm) is much greater than the film thickness (2.6 μm). Thus, the grating will be more uniform through the film when compared with another wavelength that has a higher absorption coefficient. For example, the 1/*e* absorption depth is 1.8 μm at 488 nm, and hence, the peak photo-induced change in the absorption coefficient will vary significantly as a function of thickness into the film. The diffraction efficiency was measured using the diffracted beam from one of the beams used to create the grating, it reached a maximum value of 0.033 %, and after which both beams were blocked.

The diffraction grating was probed using a 655-nm laser beam at normal incidence after an hour which is much longer than the IAB thermal relaxation time. A diffracted beam was observed that arises from periodic photobleaching of IAB that occurred when the film when exposed to the two 532-nm laser beams used to create the grating. Six hundred fifty-five nanometers was chosen as the probe wavelength because at this wavelength, the IAB absorption coefficient is zero and hence Δ*α* in Eq. 1 is zero. It is therefore possible to estimate Δ*n* from Eq. 1 using the measured first-order diffraction efficiency. We show in Fig. 4b that *η*_1_ = 0.0078 %, and from Eq. 1, we estimate that Δ*n* = 0.0014.

The IAB/APC film was exposed to a single 532-nm laser beam in the *trans*-isomer absorption band at a small angle from normal incidence with an intensity of 111 μW/mm^2^ at 61 s and continually probed using the 655-nm laser beam. This caused the diffraction efficiency to decrease to 0.0055 % as can be seen in Fig. 4b. This decrease is due to some *trans*- to *cis*-isomerization that leads to a reduction in the *trans*-isomer absorption coefficient and hence the refractive index at 655 nm. We estimate the resultant Δ*n* from Eq. 1 to be Δ*n* = 0.0012 where we find that it has reduced by 0.0002. The 532-nm laser was blocked after 455 s, and the diffraction efficiency increased back to 0.0078 % by thermal relaxation from the *cis* to the *trans*-isomer. Thus, the diffraction efficiency was reduced by ~30 % by illumination at 532 nm. This shows that the *trans*-*cis* isomerization process can be used to modulate the diffraction efficiency. Higher 532-nm intensities or lower switching wavelengths are required to further decrease the diffraction efficiency.

## Conclusions

In conclusion, we have shown that a diffraction grating can be made in an IAB/APC thin film by two-beam interference and periodic photodegradation of the IAB dye. Illumination at a later time with a single beam at 532 nm into the *trans*-isomer band that is centered ~470 nm leads to a structural transition to the *cis*-isomer and a concomitant reduction in the *trans*-isomer band absorption coefficient. This results in a decrease in the diffraction efficiency when probed at 655 nm. The diffraction grating thermally relaxes back to the original diffraction efficiency when the 532-nm laser beam is blocked. Thus, we have shown that it is possible to optically modulate the diffraction efficiency in a thin film containing a dye that displays *trans*-*cis* isomerization.
